# Pharmacist-led interventions in hematological malignancies: a systematic review of clinical, process and economic outcomes

**DOI:** 10.3389/fphar.2026.1779201

**Published:** 2026-04-21

**Authors:** Amna Mohamed, Bushra Salman, Raya Al Maskari, Khalil Al-Farsi, Mohamed A. Yassin

**Affiliations:** 1 Pharmacy Department, College of Applied Sciences and Pharmacy, University of Technology and Applied Sciences, Muscat, Oman; 2 Pharmacy Department, National Hematology and Bone Marrow Transplant Center, University Medical City, Muscat, Oman; 3 Pharmacology & Clinical Pharmacy Department, College of Medicine and Health Sciences, Sultan Qaboos University, Muscat, Oman; 4 Hematology Department, National Hematology and Bone Marrow Transplant Center, University Medical City, Muscat, Oman; 5 College of Medicine, Qatar University, Doha, Qatar; 6 National Center for Cancer Care and Research, Hamad Medical Corporation, Doha, Qatar

**Keywords:** hematological malignancies (HM), hematology-oncology patients, medication management, medication safety, pharmacist intervention, pharmacist-led interventions

## Abstract

**Background:**

Pharmacist-led interventions are increasingly integrated into hematology-oncology care to address complex pharmacotherapy and medication safety challenges. However, their impact within hematological malignancies has not been comprehensively synthesized across clinical, process-related, and economic domains.

**Methods:**

A systematic review was conducted to evaluate pharmacist-led interventions in patients with hematological malignancies across inpatient and outpatient settings. Thirty-three studies were included. Owing to substantial heterogeneity in study design, interventions, and outcome definitions, a structured qualitative synthesis was performed, with findings grouped by clinical, process-related, and economic outcomes.

**Results:**

Thirty-three studies were included, reporting clinical (*n* = 24), process-related (*n* = 23), and economic outcomes (*n* = 8). Pharmacist-led interventions most consistently improved medication management processes, including identification and resolution of drug-related problems, drug interaction management, and care coordination, with high acceptance rates of pharmacist recommendations. Several studies also reported improvements in medication adherence, toxicity management, and selected patient-reported outcomes; however, findings for disease response, survival, and hospitalization were heterogeneous and not consistently statistically significant. Economic evaluations were limited but suggested potential cost savings and favorable returns on investment associated with pharmacist involvement.

**Conclusion:**

Pharmacist-led interventions in hematology-oncology care improve medication management processes and may contribute to better adherence and medication safety. However, evidence for effects on clinical and economic outcomes remains limited and heterogeneous, highlighting the need for well-designed prospective studies using standardized outcome measures.

**Systematic Review Registration:**

https://www.crd.york.ac.uk/PROSPERO/view/CRD420251147239, identifier CRD420251147239.

## 1 Introduction

Hematological malignancies, including leukemia, lymphoma, and multiple myeloma (MM), involve complex pharmacotherapy regimens characterized by high treatment intensity, narrow therapeutic indices, frequent dose modifications, and considerable toxicity risks (Sochacka-Ćwikła et al., 2021). Over the past decade, the field has undergone rapid therapeutic innovation, with the introduction of targeted therapies, monoclonal antibodies, bispecific antibodies, and cellular therapies that have transformed treatment approaches (Sochacka-Ćwikła et al., 2021; Tang et al., 2023; Hou et al., 2021). While these advances have expanded therapeutic options and improved outcomes, they have also increased the complexity of care delivery and medication management.

Compared with many solid tumors, hematological malignancies often require urgent treatment initiation, intensive laboratory monitoring, and highly individualized dose adjustments. Patients frequently receive profoundly immunosuppressive regimens associated with severe myelosuppression and a substantial risk of infection during neutropenic periods, necessitating close monitoring and proactive supportive care (Howell et al., 2022; Nucci and Anaissie, 2017). In addition, many modern hematologic therapies introduce distinct toxicity profiles and specialized monitoring requirements; for example, chimeric antigen receptor T-cell (CAR-T) therapies and bispecific antibodies require close monitoring for immune-mediated toxicities such as cytokine release syndrome and neurotoxicity, whereas targeted agents such as Bruton tyrosine kinase inhibitors and BCL-2 inhibitors necessitate careful dose optimization and monitoring for complications including cytopenias, tumor lysis syndrome, and cardiovascular adverse events (AEs) (Hou et al., 2021; Markouli et al., 2023).

Pharmacists, particularly in oncology and hematology practice, are well positioned to address these challenges through pharmacist-led interventions that support medication optimization, toxicity monitoring, adherence support, and patient education (Banez et al., 2024; Siddiqui et al., 2023; Ochs et al., 2022). In broader oncology populations, such interventions have been associated with improvements in medication safety and treatment outcomes. For example, an integrated pharmacist-led oral chemotherapy management program reported by Muluneh et al. involving 367 patients demonstrated a substantial improvement in adherence to oral anticancer therapy, with adherence rates increasing from 74% prior to program implementation to 96% following pharmacist-led management, alongside improved clinical outcomes and earlier identification of treatment-related toxicities (Colombo et al., 2017). Other studies in mixed oncology populations have similarly reported reductions in medication errors, enhanced adverse drug event (ADE) monitoring, and improvements in patient-reported outcomes (PROs) such as quality of life (QoL) and treatment satisfaction (Xiang et al., 2025; Fentie et al., 2025).

However, the current evidence base on pharmacist-led interventions in oncology is largely derived from mixed cancer populations in which outcomes are predominantly driven by patients with solid tumors. Previous systematic reviews have therefore primarily evaluated care models and outcomes relevant to solid tumor management (Colombo et al., 2017; Xiang et al., 2025; Fentie et al., 2025). As a result, the applicability of these findings to hematological malignancies remains uncertain, given the distinctive clinical characteristics, treatment intensity, and supportive care requirements associated with hematologic cancers.

Furthermore, existing reviews frequently report heterogeneous outcome measures, apply inconsistent definitions of pharmacist interventions, and evaluate pharmacist activities as part of broader multidisciplinary care models, making it difficult to isolate the specific contribution of pharmacist-led services (Chung et al., 2019).

This systematic review is the first to focus exclusively on pharmacist-led interventions in hematological malignancies, thereby addressing gaps left by previous oncology-focused reviews. In addition, it captures real-world service models beyond randomized trials, integrating clinical, process-related, and economic outcomes, supported by a formal risk-of-bias appraisal. Economic outcomes were included to reflect real-world service evaluation and health-system priorities, despite anticipated methodological heterogeneity in economic reporting. This review seeks to determine whether, in patients with hematological malignancies receiving systemic anticancer therapy, pharmacist-led medication management interventions (compared with usual or non-pharmacist-led care) improve medication adherence and safety outcomes.

By thoroughly assessing both the strengths and limitations of the current evidence base, this review aims to support service development, inform policy decisions, and guide future research efforts to enhance care for this high-risk patient population.

## 2 Methods

### 2.1 Study design

This systematic review was conducted in accordance with the Preferred Reporting Items for Systematic Reviews and Meta-Analyses (PRISMA) guidelines (Page et al., 2021). The PRISMA checklist is provided in the Supplementary Material (Supplementary Table S1). The research question was defined using the Population, Intervention, Comparator, Outcomes, and Study Design (PICOS) framework (Table 1). The review protocol was prospectively registered in the PROSPERO database (registration ID: CRD420251147239).

**TABLE 1:** PICOS framework for eligibility criteria.

Element	Description
P (Population)	Individuals of any age diagnosed with hematological malignancies, in hospital or ambulatory care settings. No restriction was placed on geographical region
I (Intervention)	Pharmacist-led interventions aimed at optimizing hematology-oncology pharmacotherapy
C (Comparator)	Usual care, non-pharmacist-led care, or pre-intervention conditions (where applicable). Studies without comparators were also included if relevant outcomes were reported
O (Outcomes)	Clinical, process-related, and economic outcomes
S (Study Design)	Randomized controlled trials, cohort studies, case-control studies, and prospective or retrospective interventional or observational studies. Only full-text, English-language publications were included

Studies were included if they met the following criteria: (1) participants of any age diagnosed with hematological malignancies; (2) pharmacist-led interventions specifically aimed at optimizing hematology-oncology pharmacotherapy, including but not limited to prescribing oversight, therapeutic drug monitoring (TDM), medication reconciliation, formulary management, dose adjustment, patient education or counseling, and implementation of clinical guidelines; and (3) reporting of at least one relevant outcome, categorized as clinical, process-related, or economic (further details described in Section 3.6).

Eligible study designs included randomized controlled trials (RCTs), prospective and retrospective cohort studies, case-control studies, quasi-experimental and pre-post interventional studies, pilot or implementation studies, and other observational designs conducted in hospital or ambulatory care settings. Due to the limited number of controlled trials exclusively assessing pharmacist-led interventions in hematological malignancies, studies without explicit comparators, such as single-arm pre-post or descriptive implementation studies, were also included to reflect real-world practice and service delivery models. These uncontrolled designs were included for descriptive synthesis rather than causal inference, and their findings were interpreted with appropriate caution. Findings from these studies were interpreted with appropriate caution. Only full-text articles published in English were considered to ensure consistent data extraction and methodological quality assessment.

Studies were excluded if they did not involve patients with hematological malignancies, if they focused solely on palliative or end-of-life care where disease-modifying therapy was not the main objective, or if they described pharmacist activities not related to optimizing hematology-oncology treatment or lacking a clearly defined clinical role. Review articles, editorials, commentaries, and conference abstracts were also excluded from this review.

Studies that included mixed cancer populations were deemed eligible only if outcomes specific to hematological malignancies were explicitly reported or could be reliably determined through subgroup analyses, tables, or Supplementary Material. Studies were excluded if hematology-specific outcomes could not be confirmed. Inclusion in the final synthesis was limited to studies that provided sufficient outcome data and methodological detail to allow for meaningful interpretation.

### 2.2 Data sources and search strategy

A systematic literature search was conducted in PubMed, Scopus, and Web of Science from inception to 1 August 2025. Search terms included variations of three core concepts: pharmacists (“pharmacist,” “clinical pharmacy,” “pharmacist-led,” “pharmacy-led,” “medication management”), hematological malignancies (“hematologic neoplasms,” “leukemia,” “lymphoma,” “myeloma,” “blood cancer”), and interventions (“intervention,” “optimization,” “stewardship,” “prescribing support,” “medication review,” “clinical service,” “treatment adherence”). Boolean operators (AND/OR) were applied, and search syntax was adapted for each database. The full search strategy for PubMed is presented in the Supplementary Material (Supplementary Table S2). Reference lists of included studies were manually screened to capture additional eligible records.

### 2.3 Study selection

All records were imported into Zotero for de-duplication. Two reviewers independently screened titles and abstracts against the eligibility criteria, followed by full-text assessment of potentially relevant studies. Disagreements at both screening stages were resolved through discussion and consensus between the two reviewers; when consensus could not be reached, a third reviewer adjudicated the decision. Most disagreements were resolved at the discussion stage without the need for arbitration.

### 2.4 Data extraction

A standardized extraction form was developed and pilot tested. Extracted data included: study identification (ID, author, year, title, journal, database); screening status (inclusion in full-text review, inclusion in final review, reason for exclusion if applicable); study characteristics (design, country); participant details; intervention description; pharmacist role; comparator (if any); outcomes assessed; key findings; and risk of bias score.

Corresponding authors were contacted when full texts were unavailable or when additional data were required (*e.g*., hematology-specific results from studies reporting combined solid and hematological outcomes). No imputation was performed for missing data.

### 2.5 Data analysis and risk of bias assessment

A structured qualitative synthesis was performed, grouping findings into clinical, process-related, and economic outcomes. Meta-analysis was not undertaken due to heterogeneity in study design, intervention components, outcome definitions, and measurement methods, which precluded meaningful quantitative pooling.

Although no single primary outcome was prespecified, outcomes were interpreted with greater emphasis on those most consistently reported across studies. Studies were also described by design and intervention types to explore sources of heterogeneity.

Risk of bias was assessed using the Joanna Briggs Institute (JBI) critical appraisal checklists, matched to study design: RCTs (13 items), cohort studies (11 items), case-control studies (10 items), analytical cross-sectional studies (8 items), quasi-experimental studies (9 items), case series (10 items), and economic evaluations (11 items) (Joanna Briggs Institute, 2017a; Hilton, 2024; Joanna Briggs Institute, 2017b; Joanna Briggs Institute, 2017c; Joanna Briggs Institute, 2017d; Munn et al., 2019; Joanna Briggs Institute, 2020a; Joanna Briggs Institute, 2020b). These tools were selected *a priori* because the review included multiple study designs, and the JBI framework provides design-specific appraisal checklists that can be applied consistently across heterogeneous evidence bases, including quasi-experimental and economic evaluation studies. For studies reporting both clinical and economic outcomes, separate checklists were applied. Each “yes” response received one point. As the JBI tools do not provide standardized cut-off values for overall risk of bias, percentage scores were calculated and studies were classified as low (≥70%), moderate (50%–69%), or high (<50%) risk of bias, consistent with previous JBI-based systematic reviews (Munn et al., 2019; McMenamin et al., 2023).

### 2.6 Outcome definitions and operational framework

Outcomes were categorized into three main domains: clinical, process-related, and economic; as detailed in Table 2. Clinical outcomes focused on patient health effects, process outcomes on quality and efficiency of care, and economic outcomes on financial and resource impacts, with representative measures provided for each domain.

**TABLE 2:** Summary of outcome domains, definitions, and representative measures.

Domain	Operational definition	Example outcome measures
Clinical outcomes	Patient-level health effects reflecting changes in treatment exposure, tolerability, disease control, or patient-reported health status attributable to the intervention	• Medication adherence and persistence • Adverse drug events and treatment-related toxicities • Treatment interruptions or discontinuations • Disease response • Progression-free survival • Overall survival • Symptoms control • Quality of life • Patient-reported outcomes • Achievement of therapeutic drug levels • Hospitalization/readmission rates
Process outcomes	Measures of healthcare delivery quality, safety, and coordination, reflecting how medications and care processes are managed within clinical workflows	• Drug-related problems identified/resolved • Pharmacist intervention frequency • Acceptance rate of pharmacist recommendations • Timeliness and completeness of laboratory monitoring • Rates of therapeutic drug monitoring • Adherence to treatment or prophylaxis guidelines/protocols • Documentation quality • Multidisciplinary coordination • Transitions-of-care processes and discharge planning
Economic outcomes	Financial or resource utilization impacts of the intervention, including both direct and indirect costs to the healthcare system, patients, and payers	• Cost savings or cost avoidance • Cost-benefit or return-on-investment estimates • Length of hospital stay • Avoided hospitalizations/readmissions • Medication-related cost reductions • Financial impact of adverse event prevention • Cost per quality-adjusted life year gained (when reported)

To note, hospitalization-related outcomes were classified as clinical when reported as safety or toxicity endpoints, and as economic outcomes when analyzed in terms of healthcare utilization or cost impact. When reporting economic outcomes, all “$” denotes USD unless otherwise stated.

## 3 Results

### 3.1 Study selection

A total of 243 records were identified through database searches: PubMed (*n* = 33), Scopus (*n* = 158), and Web of Science (*n* = 52). After de-duplication, 197 unique records remained. Title and abstract screening excluded 87 records, leaving 110 articles for full-text review. Of these, 31 met the inclusion criteria. Manual reference screening identified an additional two studies, resulting in 33 articles included in the final review. Authors of potentially eligible studies were contacted to request missing full texts or hematology-specific data, but no additional information was obtained.

**FIGURE 1:**
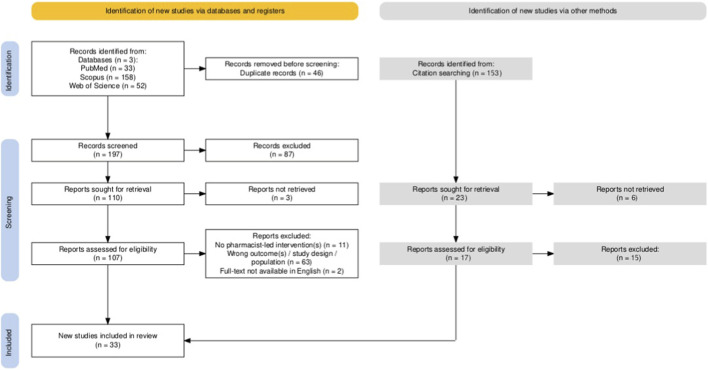
PRISMA flow diagram illustrating study selection, created using the PRISMA2020 Shiny app (Ha et al., 2022).

The study selection process is illustrated in the PRISMA flow diagram (Figure 1).

### 3.2 Study characteristics

The 33 included studies, published between 2008 and 2025, were conducted across 12 countries. Study designs were heterogeneous, with non-randomized designs predominating. Quasi-experimental studies were most common (*n* = 15, 45.5%), followed by prospective observational studies (*n* = 6, 18.2%) and cohort studies (*n* = 4, 12.1%). Retrospective observational designs were reported in two studies (6.1%), while the remaining designs were each reported in a single study.

Sample sizes ranged from 22 to 1,820 participants. Most studies focused on adults with hematological malignancies, particularly MM (*n* = 9, 26.5%), chronic myeloid leukemia (*n* = 7, 20.6%), and chronic lymphocytic leukemia (*n* = 4, 11.8%). Three studies (8.8%) included mixed cancer cohorts but reported hematology-specific outcomes separately.

Pharmacist-led interventions were grouped into three non-mutually exclusive categories, as several studies reported multiple intervention components. Medication review and optimization was the most common intervention type (*n* = 23, 69.7%). Patient education, counseling, and adherence support were reported in 22 studies (66.7%). Clinical and service integration was described in 14 studies (41.2%).

Comparators also varied. Twelve studies (35.3%) were single-arm evaluations without a comparator. Eleven (32.4%) used pre-intervention baselines. Five (15.2%) compared outcomes with historical, retrospective, or concurrent usual-care cohorts. Three (8.8%) reported active comparators, such as traditional physician-managed clinics or patients receiving identical monitoring tools without pharmacist follow-up. Two (5.9%) used risk-stratified or within-patient comparisons. A detailed overview of study characteristics is provided in Table 3.

**TABLE 3:** Characteristics of the included studies (*n* = 33).

First author	Publication year	Study design	Country	Population	Intervention category	Intervention description	Comparator
Tan et al. (2021)	2021	Prospective observational descriptive study	Malaysia	65 adult CML patients on long-term TKI therapy	Medication review and optimization/Patient education and adherence support	Medication optimization over 6 months with counselling, medication review, DRP resolution, adherence aids, and follow-up calls	None
Zerbit et al. (2022)	2022	Prospective observational study with economic evaluation	France	180 adult patients with MM, CLL, CML receiving oral anticancer therapy	Medication review and optimization/Patient education and adherence support	Medication review, DDI reduction, education, adherence support, TDM, and telemonitoring	None
Zerbit et al., 2020	2022	Observational cohort study	France	155 adult patients with B-cell hematological malignancies receiving ibrutinib (*n* = 42 in program vs. 113 usual care)	Medication review and optimization/Patient education	Multimodal PCP with scheduled consultations focusing on oral targeted therapy	Usual care cohort (no PCP)
Mauro et al. (2019)	2019	Randomized pilot trial (single-blinded)	United States	40 adult MM patients new to lenalidomide therapy	Patient education and adherence support	Intervention group received SPB with real-time alerts and pharmacist follow-up if bottle uncapping adherence <80%	Control group using inactive SPB (no alerts, no pharmacist follow-up)
Périchou et al. (2020)	2020	Quasi-experimental pre-post intervention study	France	50 adult MM outpatients receiving IMiDs	Patient education and adherence support	One-hour group education session on managing hematological and thromboembolic toxicities	Pre-implementation of the educational session
Farias et al. (2016)	2016	Quasi-experimental pre-post intervention study	Brazil	Inpatients and outpatients with hematological diseases (13,565 prescriptions)	Medication review and optimization	CPS reviewing prescriptions, patient labs, and protocol compliance	Pre-implementation period without CPS
Albayrak and Özbalci (2024)	2020	Prospective observational study	Turkey	140 hospitalized patients with hematological malignancies	Medication review and optimization	Comprehensive medication reviews using PCNE v9.1 to identify and resolve DRPs	None
Moulin et al. (2017)	2017	Quasi-experimental pre-post intervention study	Brazil	23 CML patients receiving TKIs	Patient education and adherence support	Scheduled adherence assessments and quality of life questionnaires	Non-monitored patients receiving standard care
Virani et al. (2020)	2020	Retrospective observational study	United States	241 MM outpatient consults	Clinical and service integration/Medication review and optimization/Patient education and adherence support	Pharmacist integrated into multidisciplinary team providing dose adjustments, education, and medication reconciliation	None
Ghasoub et al. (2024)	2024	Retrospective cohort comparison before-and-after clinic implementation	Qatar	22 MM patients	Clinical and service integration/Patient education and adherence support	Dedicated MM clinic managing supportive medications, labs, refills, adherence, and education	Pre-clinic baseline cohort (prior to clinic launch)
Knight et al. (2022)	2022	Prospective implementation study (quasi-experimental)	United States	107 adult patients diagnosed with a hematological malignancy ≥3 months	Clinical and service integration	Systematic multidisciplinary intervention delivered to patients who screened positive for financial toxicity; pharmacist-based intervention was focused on patient medications and potential cost savings/compliance	Pre-intervention baseline values (for QoL outcomes) and a non-intervention cohort of similar high-risk patients (for survival)
Ali et al. (2021)	2021	Prospective observational study	Pakistan	1,820 pediatric patients receiving chemotherapy, immunosuppressants, iron chelation, or post-BMT care	Clinical and service integration/Medication review and optimization/Patient education and adherence support	Ambulatory clinic pharmacist performing medication history review, dose adjustments, genomic profiling, and counseling	None
Gonçalves et al. (2025)	2025	Quasi-experimental pre-post intervention study	Brazil	Primary caregivers of 29 pediatric patients with ALL receiving chemotherapeutic agents	Patient education and adherence support	Medication therapy management with personalized consultations and educational booklets	Pre-intervention baseline (no caregiver education)
de Boer et al. (2025)	2025	Retrospective chart review	Canada	88 adult CLL patients starting venetoclax	Clinical and service integration/Medication review and optimization/Patient education and adherence support	Venetoclax ramp-up clinic managing TLS risk, titration, education, interactions, and adverse events	None
Santoleri et al. (2019)	2019	Quasi-experimental pre-post intervention study	Italy	123 adult CML patients on imatinib, nilotinib, or dasatinib	Patient education and adherence support	Treatment diary and drug data sheet with pharmacist counseling at drug dispensation	Within-patient comparison of adherence during periods with diary use vs. without diary use (person-time analysis)
Devaux et al. (2022)	2022	Prospective case-control study	France	114 adults starting first hematotoxic treatment for HL/NHL (78 matched case/control population)	Clinical and service integration/Patient education and adherence support	Multidisciplinary program (UMACOACH) with structured pharmacist consultation and supervised nurse calls	Retrospectively matched usual-care patients without pharmacist-guided follow-up
Lam and Cheung (2016)	2016	Retrospective comparative cohort study	United States	56 adult CML patients (chronic/accelerated/blast) on TKIs	Medication review and optimization/Patient education and adherence support	Longitudinal care monitoring adherence, side effects, interactions, TKI dose adjustments, labs, and counseling	Usual care group from a prior retrospective study (Campbell, 2012)
Wyatt et al. (2025)	2025	Retrospective cohort study	United States	145 adult CLL/SLL patients receiving oral oncolytics	Medication review and optimization/Patient education and adherence support	Specialty pharmacist monitoring adherence, persistence, discontinuation, switching, and adverse effects	None
Larbre et al., 2025	2025	Prospective observational cohort study	France	83 adult outpatients on ibrutinib (Oncoral program)	Clinical and service integration/Medication review and optimization/Patient education and adherence support	Multidisciplinary outpatient follow-up with scheduled interviews and pharmacist/nurse interventions (addressing AEs, DDIs, medication intake, and community-hospital coordination)	None
Reslan et al. (2020)	2020	Quasi-experimental pre-post intervention study	Australia	82 adult hematology outpatients (AML, ALL, MDS) at high risk for invasive fungal infections	Medication review and optimization	Weekly review of antifungal prophylaxis appropriateness per consensus guidelines	Pre-intervention baseline
Nguyen (2023)	2023	Retrospective observational study with cost avoidance analysis	United States	Adult cancer patients (hematology subset (CML, CLL/SLL) reported separately)	Medication review and optimization	Medication reviews with dose adjustments, interruptions, discontinuations, or resumptions of oral oncolytics	None
Mansour et al. (2024)	2024	Prospective, interventional pilot study	Algeria	130 oncology-hematology inpatients/outpatients	Medication review and optimization/Patient education and adherence support	Comprehensive medication management with DRP identification via PCNE v9.1 and adherence assessment using MMAS-8	None
de Grégori et al. (2020)	2020	Prospective observational cohort study	France	558 adults receiving first injectable chemotherapy and/or immunotherapy; hematology subset = 62% of total (*n* = 346)	Clinical and service integration/Medication review and optimization/Patient education and adherence support	Multidisciplinary program (UMACOACH) with structured pharmacist pharmacist-led interventions including medication history, validation, medication reconciliation, education, AE counseling and monitoring	None
Zanetti et al. (2023)	2023	Interventional study with historical control (quasi-experimental)	Brazil	61 allo-HSCT patients (*n* = 33 intervention group vs. *n* = 28 control group)	Clinical and service integration/Medication review and optimization/Patient education and adherence support	Pharmacist integrated into allo-HSCT team providing daily follow-up, medication reconciliation, labs, education, and post-discharge consultations	Historical control group receiving usual care, without pharmacist involvement
Wind et al. (2021)	2021	Prospective quality improvement study (quasi-experimental)	United States	28 adult patients with hematological malignancies admitted for scheduled chemotherapy	Clinical and service integration/Medication review and optimization	Transitions-of-care optimization via PDSA cycles focusing on medication accuracy, insurance, communication, and documentation	Pre-intervention baseline
Gawedzki and Collins (2021)	2021	Quasi-experimental pre-post intervention study	United States	60 adults undergoing allo-HSCT	Medication review and optimization	TDM protocol for immunosuppressants, standardizing tacrolimus dosing based on labs, organ function, and interactions	Pre-intervention usual care
Prot-Labarthe et al. (2008)	2008	Prospective descriptive study	Canada	29 pediatric HSCT patients	Medication review and optimization	DRP identification (using a SFPC) and resolution with therapeutic recommendations across multidisciplinary teams	None
Lucena et al. (2018)	2018	Retrospective quality improvement study	United States	114 adults admitted to malignant hematology/BMT services	Medication review and optimization	MRM screening with pharmacist-led histories and interventions for high-risk patients	Patients classified as “not high risk” by the MRM screening tool served as a comparator group to those classified as “high risk”
Opsomer et al. (2016)	2016	Prospective interventional cohort (quasi-experimental)	France	68 adult patients starting their first chemotherapy regimen	Patient education and adherence support	Pre-chemotherapy counseling with regimen explanation, symptom management, and follow-up	Pre-intervention status within same cohort
Muluneh et al. (2018)	2018	Quasi-experimental pre-post intervention study	United States	107 adult cancer patients on oral chemotherapy; hematology subset = 35% of total (*n* = 37). CML patients analyzed separately for molecular response outcomes	Clinical and service integration/Medication review and optimization/Patient education and adherence support	Integrated oral chemotherapy management combining specialty pharmacy and clinical services for education, adherence, AE management, dosing, and labs	Historical pre-intervention CML cohort from same institution (for molecular response rates and adherence)
Chen et al. (2020)	2020	Retrospective, quasi-experimental before-after study	Taiwan	1,443 hematology inpatients (*n* = 670 pre- vs. *n* = 773 post)	Clinical and service integration/Medication review and optimization/Patient education and adherence support	Introduction of a ward-based pharmacist participating in rounds, reviewing medications, counseling, and standardizing chemotherapy protocols	Pre-intervention usual care
Sweiss et al. (2018)	2018	Retrospective quasi-experimental study	United States	101 adult MM patients (*n* = 44 traditional physician-managed clinic vs. *n* = 57 collaborative physician-pharmacist clinic)	Clinical and service integration	Multidisciplinary MM clinic with embedded pharmacist seeing all patients per visit	Traditional physician-managed clinic (pharmacist consult on request)
Sweiss et al. (2020)	2019	Retrospective chart review (quasi-experimental)	United States	101 adult MM patients (*n* = 44 traditional physician-managed clinic vs. *n* = 57 collaborative physician-pharmacist clinic)	Clinical and service integration/Medication review and optimization	Collaborative physician-pharmacist MM clinic with full medications reconciliation, supportive care, AE management, and access coordination	Traditional physician-managed clinic (pharmacist consult on request)

Abbreviations: AE, adverse event; ALL, acute lymphoblastic leukemia; AML, acute myeloid leukemia; BMT, bone marrow transplant; CML, chronic myeloid leukemia; CLL, chronic lymphocytic leukemia; CPS, clinical pharmacy service; DDIs, Drug-Drug Interactions; DRP, Drug-Related Problem; HL, hodgkin lymphoma; HSCT/allo-HSCT, Hematopoietic Stem Cell Transplant/Allogeneic Hematopoietic Stem Cell Transplant; IMiD, immunomodulatory drug; MDS, myelodysplastic syndrome; MM, multiple myeloma; MMAS, morisky medication adherence scale; MRM, Medication-Related Morbidity; NHL, Non-Hodgkin Lymphoma; PCNE, pharmaceutical care network europe; PCP, pharmaceutical care program; PDSA, Plan-Do-Study-Act; SFPC, standardized french clinical pharmacy tool; SLL, small lymphocytic lymphoma; SPB, smart pill bottle; TDM, therapeutic drug monitoring; TKI, tyrosine kinase inhibitor; UMACOACH, Unité Médicale Ambulatoire de Cancérologie - Collaboration Assistance Chimiothérapie.

### 3.3 Risk of bias assessment

**FIGURE 2:**
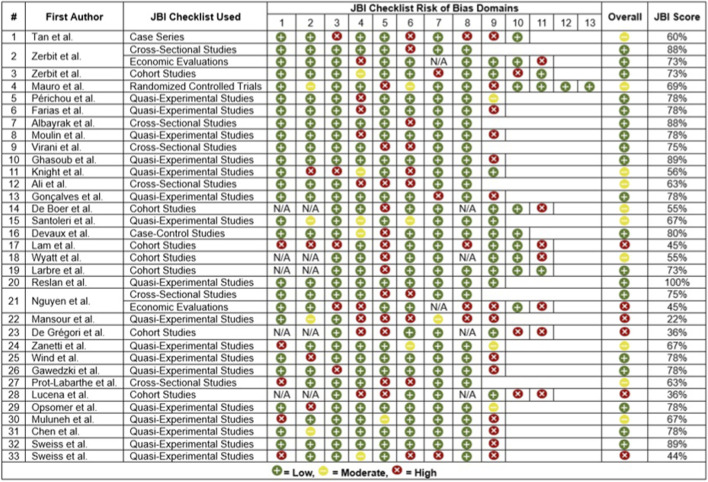
Risk of bias assessment of included studies based on the Joanna Briggs Institute (JBI) critical appraisal checklists.

Risk of bias was assessed using the JBI critical appraisal checklists matched to each study design. Two studies (Zerbit et al. (Zerbit et al., 2022) and Nguyen et al. (Nguyen, 2023)) underwent dual appraisal for observational and economic components, resulting in a total of 35 individual assessments. Of these, 19 (54.3%) were rated as low risk, 10 (28.6%) as moderate risk, and 6 (17.1%) as high risk. Figure 2 summarizes the distribution of risk of bias across included studies.

### 3.4 Synthesis of findings

Across the 33 included studies, 24 reported clinical outcomes, 23 reported process-related outcomes, and 8 reported economic outcomes. Several studies reported outcomes across more than one domain.

#### 3.4.1 Clinical outcomes

Clinical outcomes were reported in 24 studies (72.7%). Medication adherence and persistence were evaluated in 9 studies (37.5%). Several studies reported improved adherence following pharmacist involvement, including increases from 86.5% to 93.6% (*p* = 0.0007) and from 40.5% to 70.4% (*p* = 0.0115) among patients receiving oral anticancer therapies (Santoleri et al., 2019; Zanetti et al., 2023). Adherence exceeding 90% was achieved in 95% of patients receiving pharmacist-supported care compared with 73.6% in a historical cohort (*p* = 0.029) (Muluneh et al., 2018), and pharmacist monitoring was also associated with significantly higher adherence in another study (*p* = 0.0135) (Moulin et al., 2017). However, improvements were not consistently statistically significant across all studies; for example, adherence rates of 99% versus 92% in pharmacist-managed versus physician-managed care did not reach statistical significance (*p* = 0.15) (Zerbit et al., 2020). Other studies reported high adherence levels or identified non-adherence as a drug-related problem (DRP) without comparative analysis (Périchou et al., 2020; Mansour et al., 2024).

AEs and treatment-related toxicities were reported in 14 studies (58.3%). Reductions in severe toxicities were observed in some settings, including lower rates of grade ≥3 AEs (8% vs. 15%) (Zerbit et al., 2020) and grade 3-4 toxicities (26% vs. 38%, p = 0.001) (Devaux et al., 2022). Pharmacist-led TDM in transplant populations was also associated with fewer early post-transplant AEs (11 vs. 20 at day +30, *p* = 0.03) (Gawedzki and Collins, 2021). Several studies primarily reported the identification or management of adverse drug reactions (ADRs) within DRP assessments rather than demonstrating reductions in toxicity incidence (Tan et al., 2021; Farias et al., 2016; Albayrak and Özbalci, 2024; Prot-Labarthe et al., 2008). Within a similar context, treatment interruptions or discontinuations were reported in 4 studies (16.6%). AEs contributed to 36% of treatment discontinuations and 72% of therapy switches in one monitoring program (Wyatt et al., 2025), while pharmacist review resulted in discontinuation of unjustified prescriptions in 39% of cases in another study (de Grégori et al., 2020). On the other hand, pharmacist involvement was associated with longer time to treatment failure in one comparative study (*p* = 0.0005) (Zerbit et al., 2020).

Disease response, survival outcomes, and PROs were assessed in 13 studies (54.2%), with mixed findings. Improved progression-free survival (*p* = 0.002) and overall survival (*p* = 0.004) were reported in one comparative study (Zerbit et al., 2020), while increased molecular remission rates were observed among patients with chronic myeloid leukemia receiving pharmacist monitoring (Moulin et al., 2017). Improvements in PROs, including QoL and psychological coping scores, were also reported (Knight et al., 2022; Opsomer et al., 2016). However, several studies reported neutral findings. For example, improvements in major molecular response did not reach statistical significance (*p* = 0.0575) despite improved early molecular response in one study (Muluneh et al., 2018), and transplant mortality was similar between intervention and control groups in another study (*p* = 0.8029) (Zanetti et al., 2023).

Outcomes related to therapeutic drug exposure or dose intensity were reported in 5 studies (20.8%). High rates of target dose achievement were observed during venetoclax ramp-up protocols (94.3%) (de Boer et al., 2025), and monitored cohorts maintained high relative dose intensity (mean 93.7%) (Larbre et al., 2025). However, implementation of TDM protocols did not consistently improve drug concentration targets, with no difference in tacrolimus therapeutic levels observed at day +100 in one study (Gawedzki and Collins, 2021).

Hospitalization-related outcomes were reported in 3 studies (12.5%). One study documented unplanned admissions in 5.7% of patients during venetoclax ramp-up (de Boer et al., 2025), while another observed fewer rehospitalizations following pharmacist intervention, although this difference was not statistically significant (*p* = 0.217) (Devaux et al., 2022). Similarly, transplant readmission rates did not differ significantly between intervention and control groups in another study (*p* = 0.5006) (Zanetti et al., 2023).

While pharmacist involvement was frequently associated with improvements in adherence, toxicity management, and PROs, findings across clinical outcomes were heterogeneous, with both statistically significant benefits and neutral results reported across studies. Table 4 summarizes the clinical outcomes reported across included studies.

**TABLE 4:** Summary of clinical outcomes reported across included studies (*n* = 24).

First author	Medication adherence/persistence	ADEs/treatment-related toxicities	Treatment interruptions/discontinuations	Disease response/PFS/OS/Symptoms control/QoL/PROs	Therapeutic drug levels/dose intensity	Hospitalization/readmission
Tan et al. (2021)	—	ADRs reported among DRPs (45.5%)	Treatment discontinuations after monitoring (31.8%)	—	—	—
Zerbit et al. (2022)	—	—	—	PIs led to improved survival (n = 9, 1.9%)	—	—
Zerbit et al. (2020)	Adherence higher in PCP group (99% vs. 92%, *p* = 0.15)	Grade ≥3 AEs lower in PCP group (8% vs. 15%)	Longer TTF in PCP group (*p* = 0.0005)	Improved PFS (*p* = 0.002) and OS (*p* = 0.004)	—	—
Périchou et al. (2020)	Most patients (97%) achieved a MPR ≥80%	Improved patient knowledge on IMiD side effects (*p* < 0.001) at 1 months and 6 months compared to baseline	—	—	—	—
Farias et al. (2016)	—	Clinically significant DRPs detected (serious: 21; 11% in total; 1% potentially lethal)	—	71% of PI had a significant impact in the clinical pharmacist group vs. 58% in controls	—	—
Albayrak and Özbalci (2024)	—	ADRs reported among DRPs (96.7%)	—	—	—	—
Moulin et al. (2017)	Higher adherence in pharmacist-monitored group (*p* = 0.0135)	—	—	Improvement in QoL; patients reported fewer complaints and symptoms during the 4-month period (p < 0.05) Improvement in molecular parameters in CML (no improvement in control group, vs. 87.0%–95.6% improvement in monitored group)	—	—
Knight et al. (2022)	—	—	—	Improved QoL (PROMIS physical 13.7 vs. 12.5; mental 12.4 vs. 11.4; both *p* < 0.001); improved OS at 6 and 12 months (81.4% vs. 73.9%; 73.0% vs. 46.4%); survival benefit (HR 0.44, *p* = 0.017; adjusted *p* = 0.034)	—	—
Ali et al. (2021)	—	Chemotherapy complication management (14%)	—	—	Hydroxyurea dose adjustments following genomic profiling (4.8%)	—
Gonçalves et al. (2025)	—	—	—	Improved caregiver knowledge and medication administration ease (*p* < 0.001)	—	—
de Boer et al. (2025)	—	Non-TLS AEs in 45.5% of patients (mostly low grade); no clinical TLS	—	—	94.3% reached venetoclax target dose	Unplanned admissions during ramp-up (5.7%)
Santoleri et al., 2019	Higher adherence (93.6% vs. 86.5%, *p* = 0.0007) after pharmacist management of an administration diary	—	—	QoL VAS mean while on TKIs 3.46/5	—	—
Devaux et al. (2022)	—	Fewer grade 3–4 AEs (26% vs. 38%, *p* = 0.001) in the multidisciplinary monitoring program group	—	QoL improved (mean + 5.1 on EORTC scale); no difference in response or survival	ARDI of more than 85% achieved in 92% cases vs. 82% controls (*p* = 0.138)	Rehospitalizations fewer in cases but not significant (*p* = 0.217)
Lam and Cheung (2016)	Higher adherence with pharmacist management (88.6% vs. 65.8%, *p* = 0.0046)	AEs management interventions (16.8% of PIs)	—	—	—	—
Wyatt et al. (2025)	High adherence and persistence to therapy with median PDC 0.98; only 37% non-persistence	AEs resulted in 36% of therapy discontinuations and 72% of therapy switches	37% discontinued therapy; 17% switched therapy	—	—	—
Larbre et al. (2025)	—	AEs managed in 27% of interventions	—	30-month OS 73.8%; PFS 61.8%	Mean 6-month RDI 93.7% ± 11.3%	—
Mansour et al. (2024)	Non-adherence identified in 44.6% of DRPs	—	—	—	—	—
de Grégori et al. (2020)	—	—	39% discontinued therapy due to unjustified drug prescription	Clinical impact of PIs: minor (68%), moderate (20%), major (8%)	—	—
Zanetti et al. (2023)	Adherence improved (40.5%–70.4%, *p* = 0.0115) in the intervention group	—	—	Increase in knowledge (86.5%–98.4%, *p* = 0.0001); transplant mortality assessed (12.12% in intervention group vs. 14.29% in control, *p* = 0.8029)	—	Readmissions assessed (42.85% in intervention group vs. 58.33% in control, *p* = 0.5006)
Gawedzki and Collins (2021)	—	Fewer AEs at day +30 (11 vs. 20, *p* = 0.03) in the intervention group	—	—	No difference in therapeutic tacrolimus levels at day +100	—
Prot-Labarthe et al. (2008)	—	ADRs accounted for 23.8% of DRPs	—	77.9% of interventions had positive clinical impact; 3.6% major impact	—	—
Opsomer et al. (2016)	—	—	—	Improved QoL and coping scoresin the PI group; reduced helplessness (−4.8, *p* = 0.021) and anxious preoccupation (−6.7, *p* = 0.036)	—	—
Muluneh et al. (2018)	High adherence (>90%) achieved in 95% vs. 73.6% historical (*p* = 0.029)	—	—	Higher EMR (*p* = 0.0138); MMR improvement not significant (*p* = 0.0575)	—	—
Sweiss et al. (2020)	—	Reduced PIM use (75%–33%, *p* < 0.0001; RR = 0.62) in the collaborative group	—	—	—	—

Abbreviations: ADRs, Adverse Drug Reactions; AEs, Adverse Events; ARDI, average relative dose intensity; CML, chronic myeloid leukemia; DRP, Drug-Related Problems; EMR, early molecular response; EORTC QLQ-C30, European Organisation for Research and Treatment of Cancer Quality of Life Questionnaire Core 30; IMiD, immunomodulatory drug; MMR, major molecular response; MPR, medication possession ratio; OS, overall survival; PCP, pharmaceutical care program; PDC, proportion of days covered; PIs, Pharmacist Interventions; PIMs, Potentially Inappropriate Medications; PFS, Progression-Free Survival; PROs, Patient-Reported Outcomes; PROMIS, Patient-Reported Outcomes Measurement Information System; QoL, quality of life; QoL VAS, quality of life visual analogue scale; RDI, relative dose intensity; TKI, tyrosine kinase inhibitor; TLS, tumor lysis syndrome; TTF, time to treatment failure.

#### 3.4.2 Process-related outcomes

Process-related outcomes were reported in 23 studies (69.7%). Identification and resolution of DRPs or drug-drug interactions (DDIs) were reported in 10 studies (43.5%). Pharmacists identified a broad range of medication-related issues, including dosing errors, ADRs, drug interactions, and monitoring gaps. Several studies reported high resolution rates following pharmacist intervention, including resolution of 77.3% and 78.9% of identified DRPs in two studies (Tan et al., 2021; Albayrak and Özbalci, 2024). Another study reported a significant reduction in DDIs after implementation of pharmacist-led medication review, with an 81% decrease after 1 month (*p* < 0.0001) (Zerbit et al., 2020).

Pharmacist intervention frequency was reported in 17 studies (73.9%). The number of interventions ranged from 104 to 1,970 per study, depending on study design and population (Albayrak and Özbalci, 2024; de Grégori et al., 2020). Several studies reported marked increases in intervention activity following the introduction of pharmacist services. For example, implementation of a ward-based clinical pharmacist increased the intervention rate from 0.34% to 1.87% of prescriptions (*p* < 0.00001) (Chen et al., 2020), while another study reported over 1,600 pharmacist interventions during chemotherapy management (Ali et al., 2021).

Acceptance of pharmacist recommendations was evaluated in 12 studies (52.2%). Most studies reported acceptance rates exceeding 90%, including rates of 94.4%, 96.5%, and over 98% in several evaluations of pharmacist-led medication review services (Tan et al., 2021; Zerbit et al., 2022; de Grégori et al., 2020). However, variation in uptake was observed. One study reported a notably lower acceptance rate of 40% for pharmacist recommendations related to antifungal prophylaxis optimization which was attributed due to the lack of standardized communication of recommendations (Reslan et al., 2020), highlighting heterogeneity in implementation across settings.

Laboratory ordering and TDM processes were reported in 7 studies (30.4%). Pharmacists contributed to medication safety by recommending laboratory monitoring during therapy titration or treatment initiation, including laboratory monitoring in 33% and 35.3% of cases in two studies evaluating targeted therapies (de Boer et al., 2025; Lam and Cheung, 2016). Implementation of a TDM protocol also increased empiric dose adjustments from three patients before protocol implementation to 14 after implementation (*p* = 0.002) (Gawedzki and Collins, 2021). In another study, the introduction of a TDM protocol increased the number of patients receiving monitoring from none to 14 (Chen et al., 2020).

Adherence to treatment guidelines or institutional protocols was evaluated in 5 studies (21.7%). Pharmacist-led interventions were associated with improvements in several prophylaxis and supportive care practices. For example, adherence to antifungal prophylaxis guidelines increased from 31% to 54% following pharmacist intervention (*p* = 0.0344) (Reslan et al., 2020). Another study reported improvements in adherence to multiple clinical guidelines, including bisphosphonate therapy (96% vs. 68%, *p* < 0.001), influenza vaccination (76% vs. 24%, *p* < 0.001), antiviral prophylaxis (100% vs. 58%, *p* < 0.001), and venous thromboembolism prophylaxis during immunomodulatory therapy (100% vs. 83%, *p* = 0.0035) (Sweiss et al., 2018).

Documentation practices, transitions of care, and multidisciplinary coordination were reported in 12 studies (52.2%). Pharmacist involvement contributed to improved communication across care teams, coordination of specialty medication access, and structured discharge planning. Several studies described pharmacist participation in multidisciplinary teams or transplant care coordination processes (Virani et al., 2020; Ghasoub et al., 2024; Zanetti et al., 2023). Improvements in documentation and insurance communication were also reported, with one study demonstrating improvements from 0% baseline communication of insurance benefit investigations to 100% documentation for antifungal and oral chemotherapy prescriptions following pharmacist involvement (Wind et al., 2021).

Process-related outcomes demonstrated that pharmacist integration improved medication safety processes, monitoring practices, and care coordination. However, variability in intervention uptake and implementation across studies indicates that the effectiveness of pharmacist-led services may depend on institutional workflows, multidisciplinary collaboration, and local practice environments. Table 5 summarizes the process-related outcomes reported across included studies.

**TABLE 5:** Summary of process-related outcomes reported across included studies (*n* = 23).

First author	DRPs/DDIs identified or resolved	Pharmacist intervention frequency	Acceptance rate of recommendations	Laboratory order/TDM	Guideline or protocol adherence	Documentation/TOC/Coordination
Tan et al. (2021)	198 DRPs; 77.3% resolved	233 interventions	94.4%	—	—	—
Zerbit et al. (2022)	—	651 interventions	96.5%	—	—	—
Zerbit et al. (2020)	81% reduction in DDIs after 1-month (*p* < 0.0001)	—	—	—	—	Formalized information transmission during TOC in the intervention group
Farias et al. (2016)	185 DRPs; detection increased by 106.5%	—	92%–100%	—	—	—
Albayrak and Özbalci (2024)	152 DRPs; 78.9% resolved	104 interventions	96.15%	—	—	—
Virani et al. (2020)	347 discrepancies identified	242 interventions	—	—	—	Multidisciplinary communication coordination
Ghasoub et al. (2024)	—	343 interventions	100%	Increase in ordering prechemotherapy laboratory investigations (11% vs. 20%)	Increased adherence to multiple guidelines: vitamin D and calcium supplementation (68% vs. 100%, *p* = 0.02) PCP prophylaxis (76% vs. 100%, *p* = 0.08), and VTE prophylaxis during IMiD-based treatment (6% vs. 43%, *p* = 0.001).	Multidisciplinary communication coordination
Ali et al. (2021)	—	1,665 interventions	91.5%	Pharmacogenomic profiling to assess hydroxyurea treatment effectiveness (23%)	—	—
de Boer et al. (2025)	DDIs identified in 15.9% of patients	—	—	Laboratory monitoring during venetoclax titration (33%)	TLS prophylaxis optimization (82% of the patients)	—
Lam and Cheung (2016)	DDIs identified in 19.2% of patients	567 interventions	—	Laboratory work ordered/recommended during treatment (35.3%)	—	Copay assistance coordination (1.2%)
Wyatt et al. (2025)	—	141 interventions	—	Laboratory work ordered/recommended during treatment (1%)	—	—
Larbre et al. (2025)	DDIs identified in 54.5% of patients	339 interventions	—	—	—	Multidisciplinary communication and TOC coordination (18.5%)
Reslan et al. (2020)	—	153 interventions	40%	—	Antifungal prophylaxis adherence (increased from 31% to 54%, *p* = 0.0344)	—
Nguyen (2023)	—	859 interventions	—	—	—	—
Mansour et al. (2024)	879 DRPs	875 interventions	94.1%	—	—	—
de Grégori et al. (2020)	—	1,970 interventions	>98%	—	—	Multidisciplinary communication coordination
Zanetti et al. (2023)	250 DRPs	309 interventions	89.7%	—	—	Transplant care coordination through multidisciplinary rounds and formal discussions
Wind et al. (2021)	—	—	—	—	Antimicrobial prophylaxis adherence (increased from 11% to 67%)	TOC coordination (>90% success); documentation improvements in insurance communication
Gawedzki and Collins (2021)	—	—	—	TDM protocol implementation increased empiric dose adjustments (3 vs. 14, *p* = 0.002)	—	Daily tacrolimus level documentation
Prot-Labarthe et al. (2008)	—	525 interventions	93.2%	—	—	Multidisciplinary communication coordination
Lucena et al. (2018)	—	793 interventions	—	—	—	TOC coordination (4.6%)
Chen et al. (2020)	—	826 interventions: increased from 0.34% to 1.87% (*p* < 0.00001)	96% (before) vs. 98% (after) (*p* = 0.052)	TDM protocol implementation increased number of monitored patients [0 (before) vs. 14 (after)]	—	—
Sweiss et al. (2018)	—	—	—	—	Increased adherence to multiple guidelines: bisphosphonates (96% vs. 68%, *p* < 0.001), influenza vaccination (76% vs. 24%, *p* < 0.001), antiviral prophylaxis (100% vs. 58%, *p* < 0.001), and VTE prophylaxis during IMiD-based treatment (100% vs. 83%, *p* = 0.0035)	Multidisciplinary communication coordination

Abbreviations: DDI, Drug-Drug Interaction; DRP, Drug-Related Problem; IMiD, immunomodulatory drug; PCP, pneumocystis pneumonia; TDM, therapeutic drug monitoring; TLS, tumor lysis syndrome; TOC, transitions of care; VTE, venous thromboembolism.

#### 3.4.3 Economic outcomes

Economic and resource-related outcomes were reported in 8 studies (24.2%). Cost savings or cost avoidance were reported in 7 studies (87.5%). Several studies quantified substantial economic benefits associated with pharmacist-led interventions. For example, one study estimated a total value of $189,441 for pharmacist interventions during a 39-day study period, corresponding to a projected annual value of $757,764 (Virani et al., 2020). Similarly, another study reported more than $1 million in total drug cost avoidance through pharmacist-led therapy optimization and treatment discontinuation strategies (Nguyen, 2023).

Cost-benefit or return-on-investment analyses were reported in 4 studies (50%) and generally demonstrated favorable economic returns. Reported cost-benefit ratios ranged from €3.7 per €1 invested to €7.07 per €1 invested in pharmacist-led services (Zerbit et al., 2022; de Grégori et al., 2020). In another study, implementation of a ward-based clinical pharmacist increased pharmacist interventions and was associated with a marked improvement in benefit-cost ratio, from 0.77 to 3.19, alongside increased medication cost savings and avoidance (Chen et al., 2020).

Medication-related cost reductions were reported in 5 studies (62.5%). Pharmacist involvement contributed to reduced medication expenditures through therapy optimization, dose adjustments, intravenous-to-oral switches, and facilitation of patient access to financial assistance programs. For example, one study reported savings of $197,158 through pharmacist-facilitated access to manufacturer assistance programs for high-cost therapies (Knight et al., 2022), while other studies described reductions in drug expenditures through treatment discontinuation or regimen optimization (Virani et al., 2020; Nguyen, 2023; de Grégori et al., 2020; Chen et al., 2020).

The financial impact of AEs prevention was reported in 3 studies (37.5%). Prevention or reduction of ADEs contributed substantially to avoided healthcare costs in several analyses. For example, one study identified ADE prevention as a major contributor to avoided costs (de Grégori et al., 2020), while another reported increases in prevented ADEs from 58 to 230 following pharmacist integration (Chen et al., 2020).

Resource utilization outcomes were reported less frequently. Length of hospital stay was evaluated in one study (12.5%), which reported a significant reduction from 19.27 to 16.69 days following integration of a ward-based clinical pharmacist (*p* < 0.00001) (Chen et al., 2020). Avoided hospitalizations or readmissions was reported in one study (12.5%). The study observed fewer rehospitalizations in the pharmacist-supported group, although the difference did not reach statistical significance (*p* = 0.217) (Devaux et al., 2022).

Formal cost-effectiveness analysis was reported in one study (12.5%), which estimated an incremental cost-effectiveness ratio of $96.03 per percentage increase in medication adherence (Mauro et al., 2019). These findings show that while several studies demonstrated substantial cost savings and favorable economic returns associated with pharmacist interventions, economic evaluations were heterogeneous and often limited to institutional or service-level perspectives. Only a small proportion of studies conducted formal economic analyses, and few incorporated comprehensive health economic frameworks such as cost-effectiveness modeling or sensitivity analyses.

Table 6 summarizes the economic outcomes reported across included studies.

**TABLE 6:** Economic outcomes reported across included studies (*n* = 8).

First author	Cost savings/Cost avoidance	Cost-benefit or ROI	Length of hospital stay	Avoided hospitalizations/Readmissions	Medication-related cost reductions	Financial impact of AE prevention	Cost per QALY/ICER
Zerbit et al. (2022)	Net institutional benefit €539,047	Cost-benefit ratio 7.07:1	—	—	—	—	—
Mauro et al. (2019)	—	—	—	—	—	—	ICER $96.03 per 1% adherence increase
Virani et al. (2020)	$189,441 savings during 39-day study; projected $757,764 annually	—	—	—	Yes (through dose adjustments, reconciliation)	—	—
Knight et al. (2022)	$197,158 saved via medication access programs	—	—	—	Yes (free/reduced-cost medications)	—	—
Devaux et al. (2022)	Estimated savings of €81,782 from multidisciplinary program	—	—	Fewer rehospitalizations observed (not statistically significant, *p* = 0.217)	—	Yes (reduced grade 3–4 AEs associated with cost savings)	—
Nguyen (2023)	$1,018,494 total drug cost avoidance	ROI 440%	—	—	Yes (therapy optimization and TKI discontinuation)	—	—
de Grégori et al. (2020)	€175,563 cost savings; €390,480 avoided costs	Cost-benefit ratio €3.7 per €1 invested	—	—	Yes (chemotherapy regimen optimization)	Yes (ADE prevention major driver of avoided costs)	—
Chen et al. (2020)	Cost savings increased from NT$619,180 to NT$2,554,880	Benefit-cost ratio improved from 0.77 to 3.19	LOS reduced from 19.27 to 16.69 days (*p* < 0.00001)	—	Yes (intravenous to oral switches, therapy optimization; estimated 5.75-fold increase in direct medication cost savings)	Yes (ADE prevention, 58–230)	—

Abbreviations: ADE, adverse drug event; AE, adverse event; ICER, Incremental Cost-Effectiveness Ratio; LOS, length of stay; NT$, new taiwan dollar; QALY, Quality-Adjusted Life Year; ROI, return on investment; TKI, tyrosine kinase inhibitor.

Table 7 summarizes all included studies across the three outcome domains (clinical, process-related, and economic), detailing the pharmacist roles, outcomes reported, and key findings of each study.

**TABLE 7:** Summary of pharmacist roles, outcomes reported and key findings in the included studies (*n* = 33).

First author	Study design (single arm/Comparative)	Pharmacist roles	Outcomes reported	Key findings	Outcomes type	Risk of bias
Tan et al. (2021)	Single arm	Medication reviews, DRP identification/resolution, patient counselling, adherence aids, limited prescriber contact	Number/type/causes of DRPs; interventions and acceptance; ADRs; effectiveness-related DRPs; process issues (monitoring)	198 DRPs identified (median 3/patient); 96.9% of patients had ≥1 DRP. The primary cause was insufficient disease or treatment information and inadequate outcome monitoring (47.8%). Total of 233 PIs, most commonly patient education on disease and TKI related side effects (75.1%). Most interventions were accepted (94.4%) and implemented (83.7%), resulting in resolution of 77.3% of identified DRPs	Clinical + Process	Moderate
Zerbit et al. (2022)	Single arm	Comprehensive medication management, pharmaceutical consultations, coordination, adherence support, organizational evaluation	Clinical, economic and organizational impact of PIs; physician acceptance; budget impact and cost-benefit ratio	651 PIs (1.81/admission); with an overall acceptance rate of 96.5% by physicians. 16.9% had major clinical impact and 1.9% had an impact on survival. Net institutional benefit of €539,047 was calculated with a cost-benefit ratio of 7.07:1	Clinical + Process + Economic	Low
Zerbit et al. (2020)	Comparative	Medication review, DDI identification/mitigation, toxicity management education, adherence monitoring, transition coordination	PFS, OS, TTF, adherence rates, incidence of grade ≥3 AEs	In patients with B-cell malignancies receiving ibrutinib, PCP group had longer 30-month PFS (*p* = 0.002), OS (*p* = 0.004), and TTF (*p* = 0.0005). The incidence of grade ≥3 AEs was lower (8% vs. 15%). Adherence was higher (99% vs. 92%), although this difference was not statistically significant (*p* = 0.15). DDIs were similar between the PCP and control groups at treatment initiation (1.0 vs. 0.83 DDIs/patient, respectively). After 1-month, PCP group had fewer DDIs (0.19/patient, 81% reduction). Control group had nearly the same number of DDIs as at initiation (0.77/patient, 7.2% reduction) (*p* < 0.0001)	Clinical + Process	Low
Mauro et al. (2019)	Comparative	Adherence monitoring and counselling; patient contact on non-adherence	Medication adherence (% uncapping events), cycles completed, patient satisfaction, ICER.	Median adherence 100% (intervention) vs. 87.4% (control), *p* = 0.001; intervention group completed more cycles (3.38 vs. 2.88); ICER $96.03 per % adherence increase; 60% rated service very positively	Clinical + Economic	Moderate
Périchou et al. (2020)	Comparative	Structured patient education and self-management materials for IMiD side effects	Patient knowledge (before the session then 1 and 6 months after), adherence (MPR)	Knowledge improved significantly at 1 month (*p* < 0.001) with some decline at 6 months but more patients achieved satisfactory skills (*p* < 0.01); most patients had MPR ≥80%	Clinical	Low
Farias et al. (2016)	Comparative	Antineoplastic prescription validation and interventions with prescribers/teams	DRP detection/resolution, clinical impact, types of interventions	Study conducted across two periods (A and B). Overall, CPS implementation increased DRP detection by 106.5%. 185 DRPs detected; majority were clinically significant (71% post-implementation vs. 58% pre-implementation). In both periods (A and B), the main PIs were related to dose adjustment (25% vs. 35%, respectively) and withdrawal of medication (40% vs. 33%, respectively). 100% of PIs were accepted in period A, and 92%, in period B	Clinical + Process	Low
Albayrak and Özbalci (2024)	Single arm	DRP identification and multidisciplinary collaboration; pharmacist interventions	Number/type of DRPs, interventions, acceptance rate	104 PIs; 96.15% accepted and fully implemented. 49.3% of patients had ≥1 DRP, with 152 DRPs identified overall, of which 78.9% were resolved. Possible or actual ADEs (96.7%) were the most common DRPs, primarily caused by inappropriate drug choice (94.7%), particularly inappropriate drug combinations (93.4%). Potential DDIs were identified in 30.7% of patients at C risk, 7.9% at D risk, and 4.3% at X risk	Clinical + Process	Low
Moulin et al. (2017)	Comparative	Adherence counselling, TKI monitoring, tailored education	Adherence, patient-reported symptoms, QoL scores, early clinical condition	Treatment adherence was higher in pharmacist-monitored patients compared with non-monitored patients (*p* = 0.0135). PI reduced the average number of patients reporting any symptoms/complaints from 3 to 1. Pharmacist accompaniment was associated with increased molecular remission in CML patients: no improvement in molecular parameters observed in the control group, whereas monitored group showed improvement rates of 87.0%–95.6% after 4-months of follow-up	Clinical	Low
Virani et al. (2020)	Single arm	Board-certified oncology pharmacist led 474 documented interventions across 241 consults. Actions included medication teaching, dose optimization, pain management, reconciliation, chemotherapy management	Types, volume and economic valuation of interventions; discrepancies resolved; projected savings	The most frequent interventions made were: medication teaching (*n* = 97), dose adjustments (*n* = 82), and medication reconciliation (*n* = 63, 347 discrepancies found which accounts for 6 discrepancies/reconciliation). The value of interventions during the 39-day study period was $189,441; projected $757,764/year	Process + Economic	Low
Ghasoub et al. (2024)	Comparative	Advanced hematology pharmacists reviewed therapy, reordered supportive meds, monitored efficacy/safety, conducted reconciliation, education, managed drug interactions, referred subspecialties, issued refills, identified ADRs, and dose-adjusted treatments	Number of interventions (pre vs. post), time to bisphosphonate initiation, rates of appropriate VTE prophylaxis, vitamin D/calcium supplementation, number of refills and lab orders	Post clinic implementation: interventions increased significantly (76 vs. 343, *p* = 0.004); bisphosphonate initiation improved dramatically (median 14 vs. 206 days, *p* = 0.008); appropriate VTE prophylaxis rose from 6% to 43% (*p* = 0.001); appropriate PCP prophylaxis rose from 76% to 100% (*p* = 0.08); appropriate vitamin D and calcium supplementation reached 100% vs. 68% (*p* = 0.02). Pharmacists performed 343 interventions across 73 visits (median 4 visits/patient), achieving 100% acceptance rate	Process	Low
Knight et al. (2022)	Comparative	Medication/cost assessment, access facilitation, manufacturer assistance, grant funding coordination	Cost savings, OS, QoL	Obtained free/reduced medications thus saving $197,158 USD (range $29,909-$639,801). Intervention was associated with improved QoL, with higher PROMIS physical (13.7 vs. 12.5) and mental health scores (12.4 vs. 11.4) compared with baseline (both *p* < 0.001). Mortality was also lower (27% vs. 43%). Estimated OS at 6 and 12 months was higher in the intervention group (81.4% vs. 73.9%; and 73.0% vs. 46.4%). Intervention associated with improved survival on univariate analysis (HR 0.44, *p* = 0.017) and after multivariable adjustment (HR 0.44, *p* = 0.034)	Clinical + Economic	Moderate
Ali et al. (2021)	Single arm	Direct interviews, medication history review, pharmacogenomic-guided interventions, supportive care and chemotherapy dose adjustments, patient/caregiver education	Number/type of interventions; inferred impact on AEs and readmissions	1,665 interventions in total. This included: review of medication histories (24%), deferiprone dose adjustments (24%), genomic profiling for beta thalassemia patients undergoing hydroxyurea to assess whether treatment was clinically effective or not (23%), management of chemotherapy-related complications (14%), and patient education and counseling (6.6%). 91.5% of interventions accepted	Clinical + Process	Moderate
Gonçalves et al. (2025)	Comparative	Caregiver-targeted health education and administration support	Caregiver-reported ease of medication administration, pharmaceutical knowledge, understanding drug importance	No caregivers reported extreme difficulty in administration post-intervention (from 10.3% baseline); significant improvement in administration ease (*p* = 0.047), and caregiver knowledge (*p* < 0.001); improved understanding of medication importance (*p* = 0.053)	Clinical	Low
de Boer et al. (2025)	Single arm	Independent venetoclax assessment/titration, TLS prophylaxis modification, interaction/AE management, education and lab monitoring	Intervention frequency/types; TLS incidence; dose achievement; unplanned admissions	94.3% reached target dose; no clinical TLS, 2 laboratory TLS; 45.5% had non-TLS AEs (mostly low-grade); pharmacists independently modified therapy in 15.1% of visits and prevented early ramp-up complications. Drug interactions were identified in 15.9% of patients	Clinical + Process	Moderate
Santoleri et al. (2019)	Comparative	Counseling, diary and refill monitoring, tailored adherence support	Adherence (diary RDD/PDD, self-report), treatment-related QoL, reasons for non-adherence	Adherence higher with diary (RDD/PDD mean 93.6% vs. 86.5%, *p* = 0.0007); patient-reported adherence 97.4%; main non-adherence cause was forgetfulness; QoL VAS mean 3.46/5	Clinical + Process	Moderate
Devaux et al. (2022)	Comparative	Medication guidance, toxicity management and supportive therapy coordination during clinic consultation	Incidence of grade 3–4 AEs, number of re-hospitalizations, ARDI, treatment response/survival, QoL, cost savings	Significantly fewer grade 3–4 AEs in ULP group (26% vs. 38%, *p* = 0.001); ARDI > 85% achieved by 92% vs. 82% (*p* = 0.138); rehospitalizations were fewer (76 vs. 88 cases, *p* = 0.217); estimated cost savings €81,782 in favor of ULP group; QoL improvement observed (mean increase of 5.1 points on EORTC QoL scale after treatment in ULP patients). No differences in treatment responses and survival	Clinical + Economic	Low
Lam and Cheung (2016)	Comparative	Refill-based adherence monitoring (MPR), side-effect management, interactions detection, lab ordering, patient education and co-pay assistance	Adherence (MPR ≥ 90%), pharmacist interventions, therapeutic response	Patients managed by pharmacists had higher imatinib adherence compared with usual care (88.6% vs. 65.8%, *p* = 0.0046). 567 PIs documented (mean 10.1/patient); most commonly laboratory monitoring (35.3%), DDI detection (19.2%), AEs management (16.8%), TKI dose adjustments (14.5%), non-CML drug selection (13.1%), and copay assistance (1.2%)	Clinical + Process	High
Wyatt et al. (2025)	Single arm	Chart review, counselling, continuity via integrated specialty pharmacy model; intervention delivery	Adherence (PDC), persistence, discontinuation/switching, intervention outcomes	Median PDC 0.98; 37% non-persistence with median time 10 months; pharmacists delivered 141 interventions for 69 patients (43% received interventions). Interventions outcomes included: resolving identified issues (56%), scheduling follow-up care (6%), holding medication administration (1%), adjusting dose (3%), or discontinuing medication (3%)	Clinical + Process	Moderate
Larbre et al. (2025)	Single arm	Medication review, DDI/AEs identification, administration advice, hospital-community coordination	RDI over time, DDIs/AEs prevalence, % patients needing intervention, OS/PFS at 30 months	86.7% patients received ≥1 pharmacist-nurse intervention. Interventions focused on DDIs (54.5%), management of AEs (27.0%), and community-hospital coordination (18.5%). DDIs identified in 39% of patients (major DDI in 10 patients); mean 6-month RDI 93.7% ± 11.3%; 30-month OS 73.8% and PFS 61.8%	Clinical + Process	Low
Reslan et al. (2020)	Comparative	Chart reviews, prophylaxis suitability checks, DDI assessment, recommendations to hematologists	Appropriate antifungal prophylaxis use, pharmacist recommendations and uptake, interactions identified	Appropriate guideline adherence increased from 31% to 54% (OR 2.44, *p* = 0.0344); in AML subgroup, appropriate use rose from 13% to 46% (*p* < 0.01); pharmacists made 153 recommendations (uptake 40%); one serious azole-chemotherapy interaction avoided	Process	Low
Nguyen (2023)	Single arm	Clinical decision-making for oral hematology-oncology therapy optimization including DMR-based TKI discontinuation	Direct drug cost avoidance; number/type of interventions; oncologist satisfaction; ROI.	859 interventions documented; 238 were found to have direct drug cost avoidance values. CML interventions generated $643,565 cost avoidance (average of $18,928/intervention); CLL/SLL interventions generated $374,929 (average of $13,390/intervention); high oncologist satisfaction based on internal surveys; annual ROI of 440%	Process + Economic	Low (Economic arm with high risk of bias)
Mansour et al. (2024)	Single arm	Medication review, DRP classification, proposed PIs and adherence support (MMAS)	DRP counts/types, adherence (% non-adherent), PI acceptance	879 DRP were identified (mean of 6.78 DRP/patient); 44.6% were due to non-adherence. 875 PIs (67.2% at the drug-level); 94.1% PI acceptance rate	Clinical + Process	High
de Grégori et al. (2020)	Single arm	Full-time clinical pharmacists: regimen validation, DRP resolution, counseling, supportive therapy optimization, discharge follow-up	PIs number/type/acceptance; clinical impact (CLEO tool); cost savings/avoidance; cost-benefit ratio	1,970 PIs (mean of 3.5 PI/patient); PI acceptance >98%; 68% of PIs had minor clinical impact, 20% moderate and 8% major. Overall cost savings were €175,563, with immuno- or chemotherapy regimen adjustments accounting for 6% of interventions but 84% of the savings (€148,032). €390,480 was saved through avoided costs, mainly by preventing ADEs. Net annual benefit per pharmacist €223,021; cost-benefit ratio €3.7 per €1 invested	Clinical + Process + Economic	High
Zanetti et al. (2023)	Comparative	Medication reconciliation/safety checks, daily review with labs, education, outpatient consults, adherence/knowledge tools (MedTake/BMQ)	Transplant mortality (within 100 days), GVHD, readmissions, DRPs, intervention acceptability, knowledge, adherence	250 DRPs in intervention group (mean 7.58/patient); 309 PIs (average of 9.36 interventions/patient); 89.7% of interventions accepted; 75.4% of interventions rated clinically significant; significant increase in knowledge (86.5%–98.4%, *p* = 0.0001) and adherence (40.5%–70.4%, *p* = 0.0115)	Clinical + Process	Moderate
Wind et al. (2021)	Comparative	Reconciliation, discharge counseling, insurance/pre-authorization resolution, pre-admission documentation, protocol adherence support	TOC benchmarks (documentation, specialty medication resolution), insurance communication, prescribing appropriateness, patient instructions	Implementation of PDSA cycle interventions improved TOC benchmarks from 0/11 to 7/14 (>90% success). Baseline gaps included 0% communication of insurance benefit investigations, ≤50% pre-approval resolution, high inappropriate discharge prescribing (antimicrobials 78%, antiemetics 44%), low provision of patient antimicrobial prophylaxis instructions (11%). By cycle 2, interventions (including a standardized pre-admission note, antimicrobial prophylaxis SOPs, and updated documentation) improved insurance communication to 100% for antifungals and oral chemotherapy and 67% for growth factors. Pre-approval resolution reached 67%, 100%, and 88% before admission and 100%, 100%, and 88% at discharge, respectively. By cycle 3, patient antimicrobial prophylaxis instructions improved to 67%	Process	Low
Gawedzki and Collins (2021)	Comparative	Design/implement TDM protocol, DDI reviews, empiric dosing recommendations, monitoring across continuum	Tacrolimus therapeutic levels (day +30/+100), AEs, dose adjustments, DDIs, engraftment, GVHD/relapse	No difference in therapeutic tacrolimus levels at day +100 (70% in both); fewer total AEs at day +30 post-intervention (11 vs. 20; *p* = 0.03); post-intervention group had significantly more empiric dose adjustments (*p* = 0.002) and recognition of drug interactions (*p* < 0.0001)	Clinical + Process	Low
Prot-Labarthe et al. (2008)	Single arm	DRP detection/interception, clinical recommendations (oral, phone, written, or computerized), impact estimation	Number and type of interventions, delivery mode, drug classes, documentation rate, acceptance, estimated clinical/economic impact	525 PIs made over 31 days (16.9/day). Main DRPs were ADRs (23.8%), untreated indications (17.5%), and failure to receive drug (17.0%). PIs primarily involved dose adjustments (33.1%) and drug monitoring (25.1%); with 93.2% acceptance rate. Most PIs (77.9%) had a positive clinical impact, 3.6% major, and 7.6% had negative cost impact	Clinical + Process	Moderate
Lucena et al. (2018)	Comparative	Medication histories, DRP identification during admission/hospitalization, discharge counseling	Number and type of PIs, severity and clinical value, patient characteristics linked to high-risk interventions	793 PIs; 70% classified as high risk based on following criteria: readmission within 30 days, ≥10 scheduled medications, or anticoagulation therapy. High-risk patients had more total interventions (537 vs. 256) though similar per-patient rates (6.7 vs. 7.5). Among 326 PIs of highest severity/value, most occurred during the hospital stay (305/326), with common interventions being therapeutic regimen modifications (36%), discontinuations (16%), and monitoring (16%). Patients receiving ≥5 high-severity interventions had higher rates of neutropenia (35% vs. 21%) and lower platelet counts (93 vs. 162 K/cumm)	Process	Hugh
Opsomer et al. (2016)	Comparative	Structured education and counseling, symptom recognition and self-management teaching, adherence reinforcement	Pain, fatigue, QoL (EORTC QLQ-C30), coping strategies (MAC 21), predictive factors of pain/fatigue/QoL at T3 (on the eve or day before starting the 2nd chemotherapy session), time until definitive deterioration	94% of patients completed questionnaires. At baseline, PI group had higher global QoL (61.5 vs. 51.8, *p* = 0.047), higher fighting spirit (77.8 vs. 72.3, *p* = 0.037), and lower anxious preoccupation (40.6 v.s 52.6, *p* = 0.044) compared with usual care. At T3, global QoL remained higher in the PI group (65.9 vs. 51.7, *p* = 0.017), while usual care patients had higher helpless/hopeless and anxious preoccupation scores (46.8 vs. 35.2, *p* = 0.026). Between baseline and T3, a significant decrease in helpless/hopeless was observed for all patients (−2.5, *p* = 0.003). PI group reported significantly decreased scores in helpless/hopeless (−4.8, *p* = 0.021) and anxious preoccupation (−6.7, *p* = 0.036)	Clinical	Low
Muluneh et al. (2018)	Comparative	Clinical pharmacist practitioners: pre-treatment education, adherence monitoring (self-report + MPR), AE management, collaborative modifications, lab ordering	Adherence, pharmacist interventions, AE management, molecular response (CML: EMR/MMR), patient and physician satisfaction	Malignant hematology: self-reported adherence 94.7% (MPR 93.9%). In CML, 95% achieved >90% adherence vs. 73.6% historical (*p* = 0.029); EMR achieved in 88.9% vs. 54.8% historical (*p* = 0.0138); MMR in 83.3% vs. 57.6% (*p* = 0.0575); high patient (97.8% rated education “good”/“excellent”) and physician (mean score 4.89/5 for value of pharmacist services) satisfaction	Clinical	Moderate
Chen et al., 2020	Comparative	DRP detection/resolution, TDM, reconciliation, prescribing recommendations (IV to oral switch), cost-saving strategies	Number and types of interventions, acceptance rate of recommendations; medication cost savings; cost avoidance; benefit-cost ratio; LOS.	Introduction of a clinical pharmacist increased PIs from 0.34% to 1.87% (*p* < 0.00001). The acceptance rate was not significantly different before and after clinical pharmacist involvement (96% vs. 98%, *p* = 0.052). Active pharmacist recommendations increased from 1 to 109 (*p* < 0.00001). Pharmacist involvement was associated with a 5.75-fold increase in direct medication cost savings and a rise in estimated prevented ADEs (58–230). Total cost savings and avoidance increased from NT$619,180 to NT$2,554,880, improving the benefit-cost ratio from 0.77 to 3.19. LOS reduced from 19.27 to 16.69 days	Process + Economic	Low
Sweiss et al. (2018)	Comparative	Comprehensive medication review, adherence/toxicity monitoring, supportive care optimization, REMS and insurance navigation, counselling	Adherence to supportive care guidelines, vaccination, time to initiation of bisphosphonates/PJP prophylaxis; IMiD treatment delays	Collaborative MM clinic improved adherence to guidelines, including bisphosphonate use (96% vs. 68%, *p* < 0.001) with faster initiation from diagnosis (5.5 vs. 97.5 days, *p* < 0.001) and post-ASCT reinitiation (12.5 vs. 135 days, *p* < 0.001), calcium and vitamin D coadministration (100% vs. 41%, *p* < 0.001), appropriate VTE prophylaxis during IMiD therapy (100% vs. 83%, *p* = 0.0035), antiviral prophylaxis during proteasome inhibitors therapy (100% vs. 58%, *p* < 0.001), influenza vaccination (76% vs. 24%, *p* < 0.001), and medication list provision (100% vs. 43%, *p* < 0.001). PJP prophylaxis post-ASCT initiation was faster in the collaborative clinic (11 vs. 40.5 days, *p* < 0.001). Treatment delays were reduced, with median time to IMiD initiation 7 vs. 15 days (*p* = 0.0018) and 21% vs. 85% of patients experiencing delays (*p* < 0.001)	Clinical + Process	Low
Sweiss et al. (2020)	Comparative	Medication reconciliation, deprescribing duplicates/PIMs, supportive care optimization, REMS/insurance navigation, patient education	Number of medications, polypharmacy (total and non-MM related), PIM use, supportive care adherence	Collaborative MM clinic reduced median total medications (9–7, *p* = 0.045) and non-MM meds (7–3, *p* < 0.0001); lowered polypharmacy of non-MM meds (71%–33%, *p* = 0.0003); reduced PIM use (75%–33%, *p* < 0.0001; RR = 0.62); improved provision of medication lists (43%–100%)	Clinical	High

Abbreviations: ADR, adverse drug reaction; ADE, adverse drug event; AE, adverse event; AML, acute myeloid leukemia; ARDI, average relative dose intensity; ASCT, autologous stem cell transplantation; BMQ, tool, Brief Medication Questionnaire; CBA, Cost-Benefit Analysis; CLEO, tool, Clinical, Economic, and Organisational tool (used to assess pharmacist intervention impact); CLL/SLL, Chronic Lymphocytic Leukemia/Small Lymphocytic Lymphoma; CML, chronic myeloid leukemia; CML: EMR/MMR, Early Molecular Response/Major Molecular Response (CML, outcomes); CPS, clinical pharmacy service; DDI, Drug-Drug Interaction; DMR, deep molecular response; DRP, Drug-Related Problem; EORTC QLQ-C30, European Organisation for Research and Treatment of Cancer Quality of Life Questionnaire Core 30; GVHD, Graft-Versus-Host Disease; ICER, Incremental Cost-Effectiveness Ratio; IMiD, immunomodulatory drug; IV, intravenous; LOS, length of stay; MAC, 21, Mental Adjustment to Cancer Scale 21; MM, multiple myeloma; MMAS, morisky medication adherence scale; MPR, medication possession ratio; OR, odds ratio; PCP, pharmaceutical care program; PDD, prescribed daily dose; PDC, proportion of days covered; PDSA, Plan-Do-Study-Act cycle; PFS, Progression-Free Survival; PI, pharmacist intervention; PIMs, Potentially Inappropriate Medications; PJP, *pneumocystis jirovecii* pneumonia; PROMIS, Patient-Reported Outcomes Measurement Information System; QoL, quality of life; QoL VAS, quality of life visual analogue scale; RDD, received daily dose; RDI, relative dose intensity; REMS, risk evaluation and mitigation strategies; ROI, return on investment; SOPs, Standard Operating Procedures; TDM, therapeutic drug monitoring; TKI, tyrosine kinase inhibitor; TLS, tumor lysis syndrome; TOC, transitions of care; TTF, time to treatment failure; ULP, UMACOACH, lymphoma program; VTE, venous thromboembolism.

## 4 Discussion

### 4.1 Summary of main findings

This systematic review synthesized evidence from 33 studies evaluating pharmacist-led interventions in hematology-oncology care across clinical, process-related, and economic domains. Overall, pharmacist involvement was most consistently associated with improvements in medication management processes and care coordination, while effects on clinical outcomes were more heterogeneous and economic outcomes were less frequently evaluated. These findings align with previous systematic reviews in oncology that highlight the contribution of pharmacists to medication safety and adherence support (Colombo et al., 2017; Xiang et al., 2025; Fentie et al., 2025). However, considerable heterogeneity in study design, intervention models, and outcome definitions limited direct comparisons across studies and contributed to variability in reported effects.

AEs and treatment-related toxicities were among the most frequently evaluated clinical outcomes. Pharmacists commonly contributed through activities such as medication reconciliation, identification of DDIs, TDM, and proactive toxicity management. These interventions enabled earlier detection of ADRs and medication-related problems, allowing treatment adjustments before clinically significant complications occurred (Devaux et al., 2022; Gawedzki and Collins, 2021). In some studies, pharmacist involvement was associated with reductions in severe toxicities or improved monitoring practices, although many investigations focused primarily on the identification and management of medication-related problems rather than comparative reductions in AEs (Tan et al., 2021; Farias et al., 2016; Albayrak and Özbalci, 2024). As a result, while the available evidence supports a role for pharmacists in improving medication safety, the strength of this evidence varies depending on study design and outcome measurement.

Medication adherence was another commonly evaluated outcome, particularly in studies involving long-term oral therapies. Several studies reported improvements in adherence following pharmacist-led interventions, particularly when interventions incorporated patient education, ongoing monitoring, and follow-up support (Tan et al., 2021; Zerbit et al., 2020; Mauro et al., 2019; Moulin et al., 2017; Santoleri et al., 2019; Lam and Cheung, 2016; Opsomer et al., 2016; Sweiss et al., 2018; Sweiss et al., 2020). In many studies, adherence rates increased from approximately 40%–80% under usual care to 70%–100% following intervention (Zanetti et al., 2023; Mauro et al., 2019; Santoleri et al., 2019; Lam and Cheung, 2016; Muluneh et al., 2018). Pharmacists frequently provided individualized counseling on medication administration, adverse effect management, and treatment expectations, which may improve patient understanding and engagement with therapy. In addition, structured follow-up mechanisms such as clinic visits, telephone monitoring, or medication refill tracking created opportunities to identify adherence barriers and intervene early. However, adherence measures varied widely across studies and often relied on indirect metrics such as medication possession ratios or self-reported adherence, limiting comparability. Moreover, adherence remains a surrogate outcome, and the extent to which these improvements translate into sustained clinical benefit remains uncertain.

Adherence challenges are particularly relevant in hematological malignancies requiring prolonged oral therapy and frequent monitoring, including chronic myeloid leukemia, chronic lymphocytic leukemia, and MM (Stumpf and Al-Sawaf, 2024; Tam, 2021; Bonello et al., 2019). In these settings, pharmacist involvement may help mitigate treatment complexity by improving medication education, supporting regimen management, and reducing polypharmacy, as demonstrated by reductions in medication burden in collaborative clinic models (Sweiss et al., 2020). However, the durability of these effects and their impact on long-term clinical outcomes remain insufficiently studied.

Clinical outcomes beyond adherence and toxicity demonstrated mixed findings. Some studies reported improvements in disease response, survival outcomes, or patient-reported quality of life following pharmacist-led interventions, whereas others did not observe statistically significant differences between intervention and comparator groups (Moulin et al., 2017; Knight et al., 2022; Devaux et al., 2022; Muluneh et al., 2018). These inconsistencies likely reflect differences in patient populations, treatment regimens, and intervention intensity, as well as methodological limitations. Potential associations may also be confounded by disease severity, treatment selection, and healthcare system factors.

Evidence regarding treatment interruptions, pharmacokinetic optimization, and hospitalization outcomes was similarly limited and inconsistent. Pharmacist involvement facilitated activities such as dose titration, genomic-guided dosing, and TDM in some studies, but measurable improvements in pharmacokinetic endpoints or reductions in hospitalization were not consistently observed (Ali et al., 2021; de Boer et al., 2025; Gawedzki and Collins, 2021). As a result, the limited number of studies restricts the strength of conclusions that can be drawn.

In contrast to the variability observed in clinical outcomes, process-related outcomes demonstrated more consistent improvements across studies. Pharmacists frequently identified and resolved DRPs, detected DDIs, and implemented clinical interventions aimed at optimizing therapy. Several studies reported large numbers of pharmacist interventions, ranging from hundreds to thousands of interventions within relatively short study periods (Zerbit et al., 2022; Ali et al., 2021; Nguyen, 2023; de Grégori et al., 2020). DRPs were commonly identified and frequently resolved following pharmacist intervention, with reported resolution rates exceeding 75% in some studies (Tan et al., 2021; Albayrak and Özbalci, 2024). These interventions often occurred within multidisciplinary care models where pharmacists served as medication experts, supporting prescribing decisions and enhancing communication between healthcare providers. Acceptance of pharmacist recommendations by prescribers was generally high, often exceeding 90% (Tan et al., 2021; Zerbit et al., 2022; Albayrak and Özbalci, 2024; Ghasoub et al., 2024; Mansour et al., 2024; de Grégori et al., 2020; Prot-Labarthe et al., 2008). However, this observation was not uniform across studies. One study reported a substantially lower uptake rate of 40% for pharmacist recommendations (Reslan et al., 2020), highlighting variability in interprofessional integration and implementation of pharmacist interventions across healthcare settings.

From a process perspective, pharmacist involvement frequently reshaped clinical workflows within hematology-oncology care teams. Pharmacists commonly participated in multidisciplinary rounds, coordinated transitions of care, and implemented structured monitoring practices such as TDM protocols and laboratory surveillance (Farias et al., 2016; Larbre et al., 2025; Reslan et al., 2020; Wind et al., 2021; Gawedzki and Collins, 2021; Prot-Labarthe et al., 2008; Chen et al., 2020; Chen et al., 2020; Sweiss et al., 2020). In several studies, pharmacist-led initiatives introduced systematic approaches to guideline implementation, prophylaxis protocols, and supportive care management (Reslan et al., 2020; Sweiss et al., 2020). By embedding these activities into routine clinical practice, pharmacist involvement helped standardize medication management processes in complex oncology settings. However, many interventions were implemented in tertiary or academic centers with established infrastructure, which may limit generalizability to non-tertiary or resource-limited environments. In addition, several programs were implemented as pilot or early-stage service models within highly motivated clinical teams, raising the possibility of implementation bias related to early adopters (Mauro et al., 2019; Mansour et al., 2024).

Economic outcomes were evaluated less frequently and demonstrated considerable methodological heterogeneity. Several studies reported cost savings or cost avoidance associated with pharmacist interventions, often attributed to prevention of ADEs, optimization of chemotherapy regimens, and improved medication access through financial assistance programs (Virani et al., 2020; Nguyen, 2023; de Grégori et al., 2020). Pharmacists also contributed to cost reductions through therapy optimization, intravenous-to-oral medication switches, and avoidance of unnecessary medications.

In addition to direct financial metrics, some studies reported improvements in healthcare resource utilization, such as reductions in hospital length of stay or rehospitalizations. These measures often reflect improved clinical management, including better toxicity control, early identification of adverse events, and optimized supportive care. Consequently, these clinical improvements may translate into downstream economic benefits by reducing hospitalization costs and overall healthcare utilization.

Some analyses suggested favorable economic returns for pharmacist services; however, most economic evaluations relied on short-term institutional analyses or modeled cost avoidance estimates rather than comprehensive cost-effectiveness frameworks (Zerbit et al., 2022; Virani et al., 2020; Nguyen, 2023; de Grégori et al., 2020; Prot-Labarthe et al., 2008; Chen et al., 2020). The lack of standardized economic evaluation methods and long-term follow-up limits the strength of conclusions regarding the economic value of pharmacist-led interventions.

When considered collectively, the findings across clinical, process, and economic domains suggest that pharmacist-led interventions primarily influence medication management processes, which in turn may contribute to improvements in medication safety, adherence, and healthcare resource utilization. Pharmacists frequently implemented structured medication management strategies addressing multiple aspects of treatment delivery, including medication review, toxicity monitoring, dose adjustment, TDM, and patient education. These findings suggest that pharmacist interventions operate through a multifaceted approach combining medication optimization, patient education, and systematic monitoring, which together improve treatment safety and continuity of care.

Based on the available data, the strength of evidence varies across outcome categories. Process-related outcomes and medication safety measures are supported by the most consistent findings across studies, reflecting the direct role pharmacists play in medication management. Evidence for improvements in medication adherence is moderately supportive but limited by variability in adherence measurement methods and study design. In contrast, evidence linking pharmacist involvement to survival outcomes, disease response, or economic benefit remains exploratory due to the small number of studies assessing these outcomes and the predominance of observational designs.

These findings are summarized in Table 8, which categorizes the strength of evidence across outcome domains.

**TABLE 8:** Strength of evidence across outcome domains for pharmacist-led interventions in hematological malignancies.

Outcome domain	Strength of evidence	Key mechanisms of pharmacist impact	Summary interpretation
Medication safety and drug-related problem management	Strong	Medication reconciliation, drug-drug interaction screening, therapeutic drug monitoring, toxicity management, dose adjustment	Consistent improvements in identification and resolution of medication-related problems and safer prescribing practices were observed across most studies, including several comparative designs and predominantly low-to-moderate risk of bias assessments
Process-related outcomes and care coordination	Strong	Multidisciplinary team participation, clinical workflow integration, guideline implementation, monitoring protocols, transitions-of-care coordination	Pharmacist integration consistently improved clinical workflows, prescribing support, and coordination of medication management within hematology-oncology teams, with findings supported by multiple comparative and low-risk observational studies
Medication adherence and persistence	Moderate	Patient education, adherence counseling, structured follow-up, monitoring of medication refills and adverse effects	Generally favorable effects on adherence were reported, particularly in oral therapy programs; however, heterogeneity in adherence measurement tools and the predominance of non-randomized study designs limit the certainty of the evidence
Clinical outcomes (toxicity, treatment interruptions, hospitalizations)	Moderate/Emerging	Early detection of adverse events, toxicity monitoring, dose optimization, supportive care management	Some evidence suggests reductions in treatment-related toxicity and improved monitoring practices; however, findings are inconsistent and often derived from small or non-comparative studies
Survival, disease response, and quality of life	Insufficient/Hypothesis-generating	Indirect effects through adherence support, therapy optimization, and toxicity management	Evidence for survival, disease response, and patient-reported outcomes remains limited. Most studies were not designed or powered to detect these outcomes, and findings are largely exploratory or derived from observational analyses
Economic outcomes	Insufficient/Emerging	Prevention of adverse drug events, therapy optimization, medication access programs, intravenous-to-oral conversion, avoidance of unnecessary medications	Several studies reported cost savings or cost avoidance associated with pharmacist-led interventions; however, economic evaluations were heterogeneous and frequently based on short-term institutional analyses rather than comprehensive cost-effectiveness frameworks

### 4.2 Implications for practice

Despite the methodological limitations identified across the included studies, the overall body of evidence supports the integration of pharmacists into hematology-oncology care teams, particularly in roles focused on medication safety, adherence support, toxicity monitoring, and care coordination. Hematological malignancies often require complex treatment regimens involving targeted therapies, immunomodulatory agents, and supportive care medications that require ongoing monitoring and management. In this context, pharmacist-led interventions may help address common challenges such as polypharmacy, DDIs, toxicity management, and adherence to long-term oral therapies.

From a policy and health-system perspective, broader implementation of clinical pharmacy services should be supported by standardized outcome measures, integration into multidisciplinary care models, and robust economic evaluations that account for local resource constraints and system priorities (Salman et al., 2025). These findings are consistent with recommendations from professional bodies such as the American Society of Health-System Pharmacists, the European Society for Medical Oncology, and the National Comprehensive Cancer Network, which emphasize the role of oncology pharmacists in optimizing medication safety, quality, and value (Hernandez et al., 2025; Ranchon et al., 2025; Schwartz et al., 2010).

Future research should prioritize prospective, well-controlled study designs, including pragmatic or cluster-randomized trials, to better define the causal impact of pharmacist-led interventions in hematology care. The adoption of standardized outcome sets incorporating validated adherence measures, medication safety indicators, PROs, and resource utilization, with clearly defined assessment time points, would improve comparability and facilitate evidence synthesis (Chung et al., 2019; Salman et al., 2025). Further evaluation of scalability, sustainability, and long-term economic impact, particularly in non-tertiary and resource-limited settings, is needed to strengthen the evidence base for integrating pharmacists into hematology services.

### 4.3 Strengths and limitations

A key strength of this review is the detailed characterization of pharmacist roles, intervention components, and outcome domains, which provides a structured overview of pharmacist involvement in hematology-oncology care. The inclusion of both prospective and retrospective studies allowed for a broad synthesis that captures evidence from controlled evaluations as well as real-world practice settings.

Several methodological approaches were applied to support robust interpretation of the available evidence. Critical appraisal was conducted using the JBI framework with domain-level assessment, allowing the methodological quality of individual studies to be considered when interpreting findings. Conclusions were therefore framed in accordance with study design and methodological quality, with clear differentiation between exploratory and confirmatory studies. In addition, a structured narrative synthesis was used to integrate findings across heterogeneous study designs while maintaining transparency regarding study characteristics and reported outcomes.

Despite these strengths, several limitations may affect the certainty and generalizability of the findings. Randomized evidence was limited, and most included studies utilized observational or quasi-experimental designs. Blinded outcome assessment was infrequently reported, which may increase susceptibility to detection bias for some outcomes. However, the review addressed this by carefully contextualizing reported associations and avoiding causal interpretations where methodological limitations were present.

Considerable heterogeneity was observed in intervention design, outcome definitions, and follow-up duration. Rather than attempting inappropriate quantitative pooling, the narrative synthesis grouped findings by outcome domain and intervention characteristics. This approach allowed the review to capture the multifaceted nature of pharmacist-led interventions while preserving transparency in the interpretation of results.

Reporting of pharmacist training, intervention fidelity, and resource utilization was variable across studies, which may affect assessment of reproducibility and scalability. Similarly, economic evaluations frequently relied on short-term cost avoidance estimates rather than comprehensive health economic analyses. These factors were explicitly considered during interpretation, and economic findings were presented cautiously without making definitive claims regarding long-term cost-effectiveness.

Data availability influenced synthesis in some instances. A small number of studies reported limited outcome detail or combined hematological and solid tumor populations, precluding hematology-specific interpretation. Where outcome reporting was incomplete or populations were combined, findings were described cautiously, and attempts were made to obtain disaggregated data from authors.

Finally, publication bias cannot be excluded. Conference abstracts were excluded to ensure inclusion of peer-reviewed full texts with sufficient methodological detail, but this may have increased the risk of publication bias in a rapidly evolving field. The evidence base was also dominated by studies conducted in high-income healthcare systems, most of which reported favorable findings. This imbalance may overestimate intervention effectiveness and limits applicability to primary care settings, resource-limited settings, and lower- and middle-income countries.

Despite these limitations, a substantial number of studies consistently demonstrated beneficial effects of pharmacist-led interventions across several outcome domains. The consistency of these findings across diverse study designs and practice settings strengthens confidence in the overall conclusions, while highlighting the need for further high-quality controlled studies to strengthen causal inference and evaluate long-term clinical and economic outcomes.

## 5 Conclusion

This systematic review demonstrates that pharmacist-led interventions play an important role in optimizing medication management in hematology-oncology care. Across the included studies, pharmacist involvement most consistently improved medication-related processes, including identification and resolution of DRPs, management of drug interactions, adherence support, and coordination of care within multidisciplinary teams. Overall, the available evidence supports the integration of pharmacists into hematology-oncology care teams as a strategy to enhance medication safety and treatment management for patients receiving complex therapies. However, more prospective studies using standardized clinical, patient-reported, and economic outcome measures are needed to more clearly define the long-term clinical and health system impact of pharmacist-led interventions in this setting.

## Data Availability

The original contributions presented in the study are included in the article/Supplementary Material, further inquiries can be directed to the corresponding author.
