# Supplementary material for: Pharmacist-led interventions in hematological malignancies: a systematic review of clinical, process and economic outcomes
Source: Front Pharmacol. 2026 Apr 21;17:1779201. doi: 10.3389/fphar.2026.1779201 (PMC13139178; doi:10.3389/fphar.2026.1779201)
Supplement: Supplementary file 2 [file Table2.docx]

**Supplementary File 2: Electronic Search Strategy**

| **Database** | **Search Strategy** |
| --- | --- |
| **PubMed (MEDLINE)** | (pharmacist*[Title/Abstract] OR “clinical pharmacy”[Title/Abstract] OR “clinical pharmacist*”[Title/Abstract] OR “pharmacist-led”[Title/Abstract] OR “pharmacy-led”[Title/Abstract] OR “medication management”[Title/Abstract] OR “pharmaceutical care”[Title/Abstract]) AND (“hematologic neoplasms”[MeSH Terms] OR “hematological malignanc*”[Title/Abstract] OR “hematologic malignanc*”[Title/Abstract] OR leukemia[Title/Abstract] OR lymphoma[Title/Abstract] OR myeloma[Title/Abstract] OR “blood cancer*”[Title/Abstract]) AND (intervention*[Title/Abstract] OR optimization[Title/Abstract] OR stewardship[Title/Abstract] OR “prescribing support”[Title/Abstract] OR “medication review”[Title/Abstract] OR “therapy management”[Title/Abstract] OR adherence[Title/Abstract] OR safety[Title/Abstract]) |
| **Scopus** | TITLE-ABS-KEY (pharmacist* OR “clinical pharmacy” OR “clinical pharmacist*” OR “pharmacist-led” OR “pharmacy-led” OR “medication management” OR “pharmaceutical care”) AND TITLE-ABS-KEY (“hematological malignanc*” OR “hematologic malignanc*” OR leukemia OR lymphoma OR myeloma OR “blood cancer*”) AND TITLE-ABS-KEY (intervention* OR optimization OR stewardship OR “prescribing support” OR “medication review” OR “therapy management” OR adherence OR safety) |
| **Web of Science Core Collection** | TS=(pharmacist* OR “clinical pharmacy” OR “clinical pharmacist*” OR “pharmacist-led” OR “pharmacy-led” OR “medication management” OR “pharmaceutical care”) AND TS=(“hematological malignanc*” OR “hematologic malignanc*” OR leukemia OR lymphoma OR myeloma OR “blood cancer*”) AND TS=(intervention* OR optimization OR stewardship OR “prescribing support” OR “medication review” OR “therapy management” OR adherence OR safety) |

**To Note:**

**Limits applied to all databases:**

- Language: English
- Publication type: Full-text articles
- Date range: From database inception to August 1, 2025
